# Utility of indium-111 platelet scintigraphy for understanding the mechanism of thrombocytopenia associated with myelodysplastic syndromes and chronic myelomonocytic leukemia

**DOI:** 10.1186/s40164-023-00414-1

**Published:** 2023-05-30

**Authors:** Pauline Durand, Valérie Pottier, Charles Mesguich, Frédéric Debordeaux, Estibaliz Lazaro, Jean-François Viallard, Etienne Rivière

**Affiliations:** 1 Department of Internal Medicine and Infectious Diseases, Haut-Leveque Hospital, University Hospital Centre of Bordeaux, 33600 Pessac, France; 2 Radiopharmacy Department, University Hospital Centre of Bordeaux, Pessac, France; 3 Nuclear Medicine Department, University Hospital Centre of Bordeaux, Pessac, France; 4 UMR CNRS 5164, ImmunoconcEpT and FHU ACRONIM, Bordeaux University, 33000 Bordeaux, France; 5 Faculty of Medicine, Bordeaux University, 33000 Bordeaux, France; 6 INSERM U1034, Bordeaux University, 33604 Pessac Cedex, France

**Keywords:** Myelodysplastic syndrome, Immune thrombocytopenia, Indium-111 platelet scintigraphy

## Abstract

**Background:**

Thrombocytopenia occurs in 60% of patients with myelodysplastic syndromes (MDS), increasing the risk of life-threatening haemorrhage in this population of mainly old patients with comorbidities. However, data are scare regarding immune thrombocytopenia (ITP) secondary to MDS.

**Aim:**

We analyzed the utility of indium-111 platelet scintigraphy (IPS) to better characterize the mechanisms of thrombocytopenia in 21 adult patients with MDS.

**Methods:**

Adult patients with a definite diagnosis of MDS according to the international criteria who underwent IPS between 2009 and 2018 because of an increased bleeding risk were retrospectively selected. Autologous 111Indium platelet labelling was performed with a technique similar to that described previously using a standardized method.

**Results:**

Platelet lifespan ≤ 6 days identified patients with peripheral platelet destruction. Taking into account the response to ITP-directed therapies after IPS, the sensitivity, specificity, and positive and negative predictive values of IPS were 100%, 84.6%, 80%, and 100%, respectively.

**Conclusion:**

We show that IPS can be a useful tool to identify the mechanism and guide treatment of a chronic thrombocytopenia increasing the bleeding risk in patients with MDS.

**Supplementary Information:**

The online version contains supplementary material available at 10.1186/s40164-023-00414-1.

**To the Editor,**

Myelodysplastic syndromes (MDSs) and chronic myelomonocytic leukemia (CMML) are clonal haematological diseases, and thrombocytopenia with multiple underlying mechanisms occurs in up to two thirds of patients with such disorders [1, 2]. Platelet count can be very low, resulting in increased risk for bleeding and even life-threatening haemorrhage in 10–20% patients [1, 2]. Treatments to increase the platelet count have shown variable efficacy, sometimes leading to therapeutic dead-ends with repeated or clustered platelet transfusions [1, 3]. Indium-111 (111In) platelet scintigraphy (IPS) may be a useful diagnostic tool to search for increased platelet destruction in the specific setting of potentially impaired platelet production due to MDS or CMML. No platelet kinetics studies have been specifically performed in these diseases. Here, we assessed how IPS could be useful for treating patients with both MDS or CMML and refractory thrombocytopenia by better characterizing the underlying mechanisms leading to the low platelet count. Methods are explained in Additional file 1. Briefly, response was defined as recommended in immune thrombocytopenia (ITP) [4]: complete response (CR, platelets > 100 × 109/L and no bleeding); partial response (R, platelets between 30 and 100 × 109/L with a minimal doubling of initial platelet count and no bleeding); and non-response (NR, platelets < 30 × 109/L or less than twice the initial platelet count, or presence of bleeding).

Among 389 cases of IPS registered at our centre, 21 patients with MDS or CMML underwent IPS (Table 1). None had overt clinical features of autoimmune or inflammatory associated disease. Median age of patients was 72 years (57–86). Median platelet count was 45 × 109/L (10–118). The Revised International Prognostic Scoring System (R-IPSS) was assessed in all patients, with a majority of low or intermediate-1 risk. Before IPS, the unexplained mechanism of thrombocytopenia was investigated in all patients by testing their response to ITP-directed treatments: 7 patients received steroids, 7 intravenous immunoglobulin (IVIG), 2 rituximab, 2 thrombopoietin receptor agonists (TPO-RA), and 3 received dapsone: none of these treatments increased the platelet count.

**Table 1:** Characteristics of MDS/CMML patients with positive or negative IPS

	Positive IPS (*n* = 10)	Negative IPS (*n* = 11)
Sex ratio (M/W)	5/5 (1)	5/6 (0.83)
Initial platelet count (*n*, min–max)	49 (40–83)	75 (41–91)
Bleeding symptoms	1 (10%)	3 (27%)
Isolated thrombocytopenia prior to MDS/CMML	8 (80%)	6 (54%)
Interval between first thrombocytopenia and MDS/CMML in months (*n*, min–max)	16 (1–77)	20 (8–59)
Age at diagnosis of MDS/CMML (*n*, min–max)	68 (61–75)	70 (70–77)
Platelet count at diagnosis of MDS/CMML (× 10^9^/L; *n*, min–max)	42 (32–56)	59 (44–75)
Disease (MDS/CMML)		
CMML	5 (50%)	3 (27%)
RAEB	1 (10%)	4 (37%)
MDS-MLD	2 (20%)	1 (9%)
MDS-U	1 (10%)	2 (18%)
MDS-SLD	1 (10%)	1 (9%)
Bone marrow cellularity		
Decreased	0 (0%)	2 (18%)
Normal	6 (60%)	5 (45%)
Increased	4 (40%)	4 (36%)
Megakaryocytes in bone marrow		
Decreased	1 (10%)	5 (45%)
Normal or increased	9 (90%)	6 (55%)
Dysplastic megakaryocytes^a^		
Yes	7 (70%)	2 (18%)
No	3 (30%)	9 (82%)
R-IPSS		
Low	5 (20%)	4 (37%)
Intermediate 1	4 (40%)	6 (54%)
Intermediate 2	1 (10%)	1 (9%)
High	0 (0%)	0 (0%)
Presence of ANA		
No	6 (60%)	6 (54%)
Yes	4 (40%)	4 (37%)
Not performed	0 (0%)	1 (9%)
MAIPA		
Positive	1 (10%)	1 (9%)
Negative	6 (60%)	4 (37%)
Not performed	3 (30%)	6 (54%)
Platelet kinetic study		
Interval between first thrombocytopenia and IPS, months (*n*, min–max)	22 (7–37)	22 (4–41)
Sequestration pattern		
None	1 (10%)	7 (64%)
Splenic	7 (70%)	2 (18%)
Hepatic	2 (20%)	2 (18%)
Platelet lifespan (*n*, min–max)	5.5 (5–6)	7 (6.5–7)

ANA, anti-nuclear antibody; CMML, chronic myelomonocytic leukemia; F, female; M, male; MAIPA, monoclonal antibody immobilization of platelet antigens; MDS, myelodysplastic syndrome; MDS-MLD, MDS with multilineage dysplasia; MDS-SLD, MDS with single-lineage dysplasia; MDS-U, MDS unclassified; RAEB, refractory anaemia with excess blasts; R-IPSS, Revised International Prognostic Scoring System

^a^Megakaryocytes with hypolobated nuclei, or with separated nucleus, or micro-megakaryocytes

IPS was therefore performed in all patients after a median interval of 22 months (4–41) after the first exploration of thrombocytopenia. A cut-off value of 6 days was used to distinguish patients with possible peripheral destruction of platelets (platelet lifespan ≤ 6 days, positive IPS) from patients with insufficient platelet production (platelet lifespan > 6 days, negative IPS), as suggested in previous studies [5, 6]. IPS was thus considered positive in 10 patients (positive IPS group) and negative in 11 patients (negative IPS group). Notably, most patients in the positive IPS group had splenic sequestration, but none had a platelet life span < 5 days, therefore reinforcing the hypothesis that these patients may have mixed features of ITP and MDS (Table 1), and no patient in the negative-IPS group responded to previous ITP-directed therapies. After IPS, 8 patients from the positive IPS group were re-challenged with ITP-directed treatments, i.e., steroids (n = 3), rituximab (n = 2), IVIG (n = 1), dapsone (n = 1), or TPO-RA (n = 1), which led to an increase in platelet count in 3 patients (37.5%, Table 2): partial response to successive courses of IVIG for the first, sustained partial response with steroids for the two others. Among the 10 patients with positive IPS (none in the negative IPS group), a diagnosis of ITP was finally retained in 8, because the course of the thrombocytopenia was not suggestive in 2 patients. Finally, in this small cohort, sensitivity and specificity of IPS were 100% and 84.6%, respectively. The positive predictive value of IPS in the diagnostic work-up of secondary ITP in patients with MDS/CMML was 80%, while the negative predictive value was 100%.

**Table 2:** Treatment response rate in MDS/CMML patients after IPS

Treatment	Positive IPS (*n* = 10)	Negative IPS (*n* = 11)
Platelet transfusion	3/6 (50%)	6/6 (100%)
Steroids	2/6 (33%)	0/4 (0%)
IVIG	1/4 (25%)	0/4 (0%)
Dapsone	0/2 (0%)	0/2 (0%)
Rituximab	0/2 (0%)	0/2 (0%)
TPO-RAs	0/1 (0%)	0/2 (0%)
Danatrol	1/1 (100%)	0/2 (0%)
Azacitidine	1/4 (25%)	1/2 (50%)

IPS, Indium-111 platelet scintigraphy; IVIG, intravenous immunoglobulin; MDS, myelodysplastic syndrome; CMML, chronic myelomonocytic leukemia; TPO-RAs, thrombopoietin receptor agonists

IPS may be helpful for characterizing the cause of thrombocytopenia in the specific context of MDS/CMML and low platelet count or bleeding. Platelet lifespan is informative as patients with a platelet lifespan less than 6 days could benefit from ITP-directed therapies. This is important because there is currently no definitive diagnostic test for immune thrombocytopenia associated with MDS/CMML. Quantification of reticulated platelets (i.e., immature platelet fractions) has been attempted in ITP with inconclusive results [7], but did not provide insight into the mechanism of thrombocytopenia in MDS/CMML [8]. Notably, CMML was the main disease associated with peripheral destruction of platelets in our study population, as suggested previously [2, 9]. Furthermore, we showed that a bone marrow smear is probably not useful for diagnosis of the mechanism of thrombocytopenia under these conditions [10]. This study had several limitations due to its retrospective nature and the small number of patients. However, to the best of our knowledge, this is the first study to suggest the usefulness of IPS for understanding the mechanism of clinically significant thrombocytopenia in MDS/CMML according to the later clinical course. IPS could be helpful for tailoring specific treatments for MDS/CMML patients with refractory thrombocytopenia and/or bleeding signs.

## Supplementary Information

**Additional file 1.** Supplemental details on the methodology

## Data Availability

All data and materials can be obtained upon reasonable request to the corresponding author.

## Abbreviations

ANA: Anti-nuclear antibody
CMML: Chronic myelomonocytic leukaemia
F: Female
IPS: Indium-111 platelet scintigraphy
ITP: Immune thrombocytopenia
IVIG: Intravenous immunoglobulin
M: Male
MAIPA: Monoclonal antibody immobilization of platelet antigens
MDS: Myelodysplastic syndrome
MDS-MLD: MDS with multilineage dysplasia
MDS-SLD: MDS with single-lineage dysplasia
MDS-U: MDS unclassified
RAEB: Refractory anaemia with excess blasts
R-IPSS: Revised International Prognostic Scoring System
TPO-RAs: Thrombopoietin receptor agonists

## References

[1] Kantarjian H, Giles F, List A (2007). The incidence and impact of thrombocytopenia in myelodysplastic syndromes. Cancer.

[2] Jachiet V, Moulis G, Hadjadj J (2021). Clinical spectrum, outcome and management of immune thrombocytopenia associated with myelodysplastic syndromes and chronic myelomonocytic leukemia. Haematologica.

[3] Li W, Morrone K, Kambhampati S (2016). Thrombocytopenia in MDS: epidemiology, mechanisms, clinical consequences and novel therapeutic strategies. Leukemia.

[4] Rodeghiero F, Stasi R, Gernsheimer T (2009). Standardization of terminology, definitions and outcome criteria in immune thrombocytopenic purpura of adults and children: report from an international working group. Blood.

[5] Houwerzijl E, Blom N, van der Want JVD (2005). Increased peripheral platelet destruction and caspase-3-independent programmed cell death of bone marrow megakaryocytes in myelodysplastic patients. Blood.

[6] Efira A, Cauchie P, Rauis M, de Maertelaere E (1986). Platelet survival in myelodysplastic syndromes. Acta Haematol.

[7] Kuwana M, Kurata Y, Fujimura K (2006). Preliminary laboratory based diagnostic criteria for immune thrombocytopenic purpura: evaluation by multi-center prospective study. J Thromb Haemost JTH.

[8] Cybulska A, Meintker L, Ringwald J, Krause SW (2017). Measurements of immature platelets with haematology analysers are of limited value to separate immune thrombocytopenia from bone marrow failure. Br J Haematol.

[9] Dussiau C, Dupuy H, Bidet A, Sauvezie M, De-Grande AC, Boureau L (2023). Impact of mutational status and prognostic factors on survival in chronic myelomonocytic leukemia with systemic inflammation and autoimmune disorders. HemaSphere.

[10] Albayrak M, Eliacik E, Korucu B (2014). An immune thrombocytopenic purpura-like picture preceding chronic myelomonocytic leukemia: a case series and review of the literature. Int J Hematol Oncol.

