# Supplementary material for: Utility of indium-111 platelet scintigraphy for understanding the mechanism of thrombocytopenia associated with myelodysplastic syndromes and chronic myelomonocytic leukemia
Source: Exp Hematol Oncol. 2023 May 30;12:50. doi: 10.1186/s40164-023-00414-1 (PMC10228125; doi:10.1186/s40164-023-00414-1)
Supplement: Supplementary file 1 — Additional file 1. Supplemental details on the methodology [file 40164_2023_414_MOESM1_ESM.docx]

**SUPPLEMENTAL MATERIALS & METHODS**

Adult patients with a definite diagnosis of MDS or CMML according to the international criteria^6^ who underwent IPS because of increased bleeding risk between 2009 and 2018 were retrospectively selected from the database of the Nuclear Medicine Department, University Hospital Centre of Bordeaux. To assess the performance of IPS in the diagnosis of peripheral destruction of platelets associated with MDS or CMML, patients were classified according to the presence (*MDS/CMML-ITP group*) or absence of ITP (*MDS group*) according to the international criteria,^7^ and physicians reassessed the diagnosis according to the course of thrombocytopenia following specific treatment for ITP. Data from the patients’ medical charts were collected using a standardized form. Responders were patients with a complete (platelet count > 100 × 10^9^/L) or partial (platelet count between 30 and 100 × 10^9^/L) response stable over time. Autologous ^111^In platelet labelling was performed with a technique similar to that described previously^1-3^, using a standardized method.^4^ Briefly, platelet kinetics were assessed by a 5 ml blood puncture 10 minutes and 1h after the reinjection of the autologous Indium-111 labeled-platelets, and then once a day for the following days until radioactivity signal had decreased to 10%. The site of platelet destruction was identified with a gamma camera focused on the liver and spleen regions, with a global image obtained 30 minutes after the reinjection of the autologous Indium-111 labelled-platelets and then daily. Therefore, four parameters were measured: platelets lifespan in plasma, the spleen/hepatic ratio activity, the splenic capture index, and the site of platelet destruction. Platelets lifespan in plasma was considered normal if equal to 9.1 +/- 1.2 day. Platelet sequestration was classified according to the classification system proposed by Najean et al.

Median values and quartiles were used to quantitative variables, and frequency was used to describe qualitative variables. The Mann–Whitney test was used for between-group comparisons because of the small number of patients (*n* < 30). Qualitative variables were compared using the χ^2^ test and Yates correction when applicable; otherwise Fisher’s exact test was performed. Statistical analyses were performed with IBM SPSS v26 (IBM Corp., Armonk, NY, USA). In all analyses, *P* < .05 was taken to indicate statistical significance.

This study was conducted in accordance with to the 1964 Declaration of Helsinki. Our local institutional review board approved the study.

**REFERENCES**

1- Gugliotta L, Isacchi G, Guarini A, et al. Chronic idiopathic thrombocytopenic purpura (ITP): site of platelet sequestration and results of splenectomy. A study of 197 patients. Scand. J. Haematol. 1981;26(5):407–412.

2- Hawker RJ, Hawker LM, Wilkinson AR. Indium (111In)-labelled human platelets: optimal method. Clin. Sci. Lond. Engl. 1979. 1980;58(3):243–248.

3- Najean Y. The choice of tracers for platelet kinetic and scintigraphic studies. Int. J. Rad. Appl. Instrum. B. 1986;13(2):159–164.

4- Recommended methods for radioisotope platelet survival studies: by the panel on Diagnostic Application of Radioisotopes in Hematology, International Committee for Standardization in Hematology. Blood. 1977;50(6):1137–1144.
